# Traumatic abdominal wall injuries in postmortem CT

**DOI:** 10.1007/s00414-026-03837-7

**Published:** 2026-05-29

**Authors:** Dietrich Stoevesandt, Lina Woydt, Lisa-Maria Peter, Hartmut Stefani, Carolin Richter, Sarah Heinze, Marco Weber

**Affiliations:** 1 Institute for Legal Medicine, Martin Luther University Halle-Wittenberg, Halle/Saale, Germany; 2 Clinic for Emergency and Acute Medicine, Carl-von-Basedow-Klinikum Saalekreis gGmbH, Merseburg, Germany; 3 Institute of Forensic Medicine, Medical University Graz, Graz, Austria; 4 Department of Radiology, University Hospital Halle, Ernst-Grube-Straße 40, Halle/Saale, 06120 Germany

## Abstract

**Background:**

Traumatic abdominal wall hernia (TAWH) is a rare manifestation of blunt trauma, usually linked to high-energy mechanisms such as motor vehicle collisions. While uncommon in clinical series, its true frequency in fatal trauma is poorly understood. This study aimed to determine the incidence of TAWH in postmortem computed tomography (pmCT) of traffic fatalities and to evaluate associated injury patterns, with particular focus on motor vehicle occupants (MVO).

**Methods:**

We retrospectively reviewed traffic fatalities between January 2011 and July 2023 at a single forensic institute. Of 411 cases, 218 underwent diagnostic pmCT and were systematically assessed for TAWH, defined as disruption of abdominal wall musculature or fascia. Demographic data, seatbelt status, accident details, and associated injuries were recorded. Statistical analyses included Chi-square and Mann-Whitney U tests.

**Results:**

TAWH was identified in 45 of 218 cases (20.6%), substantially higher than the 0.17–1.5% reported in clinical trauma cohorts. Within the MVO subgroup (*n* = 72), 14 cases (19.4%) demonstrated TAWH. All cases with documented restraint status occurred in seatbelted occupants (7/30). Strong correlations were found with sternal fractures, serial rib fractures, lumbar transverse process fractures, and sacroiliac joint injuries. No significant associations were observed with age, sex, or seating position.

**Conclusion:**

TAWH is a significant indicator of fatal high-energy trauma and is much more prevalent in postmortem examinations than in clinical settings. Its association with injury patterns characteristic of seatbelt loading suggests diagnostic relevance in restrained occupants. Awareness of TAWH may improve recognition of severe trauma mechanisms, and further biomechanical research is needed to clarify thresholds and mechanisms.

## Introduction

### Seatbelt safety

Fatal injuries to drivers and front seat occupants can be reduced by 40– 50% and to rear seat occupants by 25–75% when seat belts are worn by passengers in motor vehicle collisions [1]. Nevertheless, seat belts have also long been associated with certain injuries. The first injury was described in 1948 by Chance et al. This lumbar fracture dislocation of the lumbar spine occurs when the lap belt acts as fulcrum and the occupant’s spine is subjected to a combination of hyperflexion and axial tension [2].

### TAWH

**Fig. 1:**
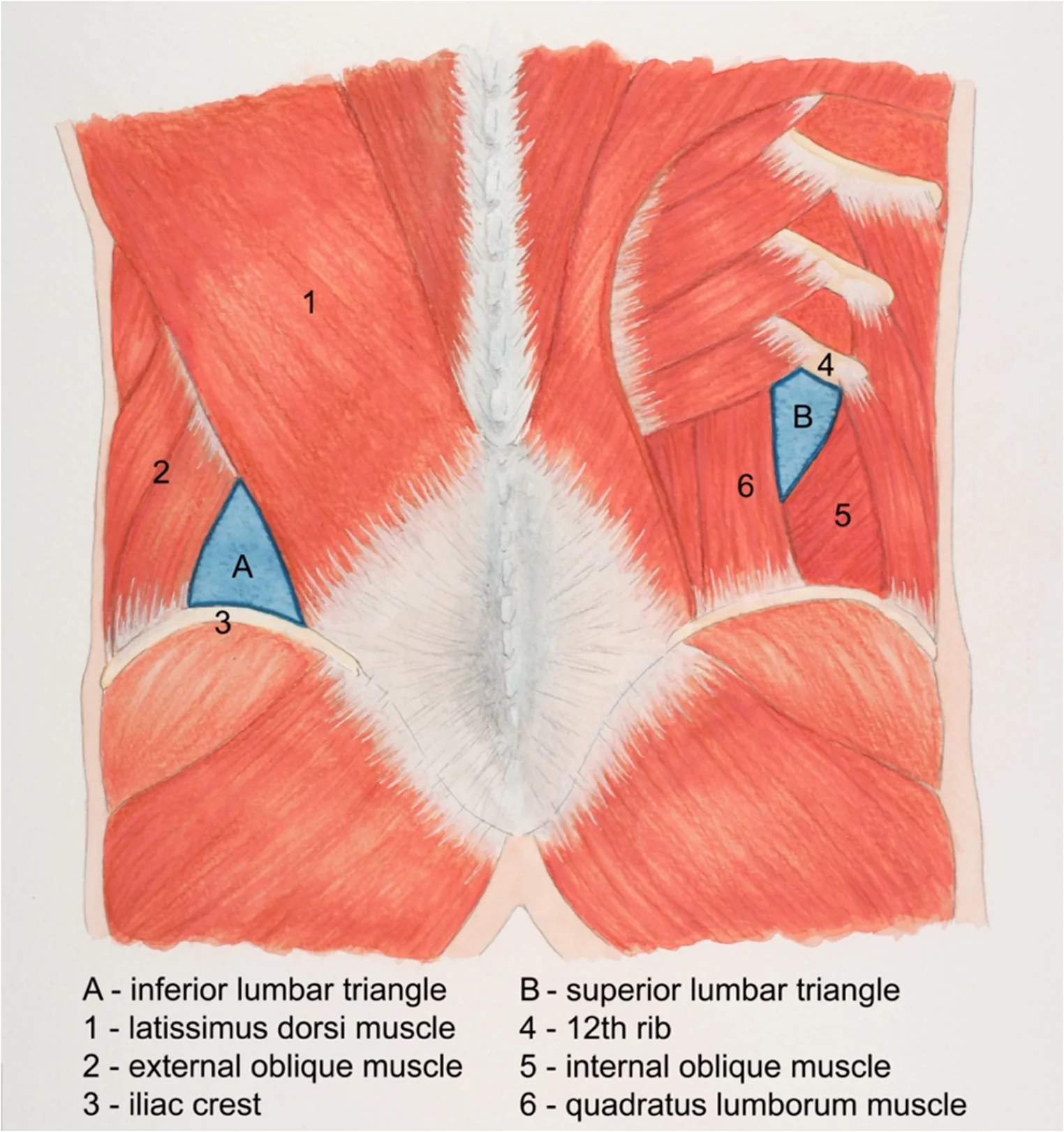
Drawing of the anatomy of the dorsal muscular wall with the inferior (A) und superior lumbar triangle

Traumatic abdominal wall hernias (TAWH), including lumbar hernias, may occur within the superior or inferior lumbar triangle. Both triangles represent areas of relative weakness in the posterolateral abdominal wall. The superior lumbar triangle (Grynfelt-Lesshaft triangle) is an inverted triangle bordered superiorly by the 12th rib, anteriorly by the internal oblique muscle, and posteriorly by the erector spinae muscle. The latissimus dorsi muscle forms the roof of the triangle, and the aponeurosis of the transversalis muscle forms the floor. The inferior lumbar (Petit’s) triangle is an upright triangle bordered inferiorly by the iliac crest, anteriorly by the external oblique muscle, and posteriorly by the latissimus dorsi muscle. Superficial fascia and skin constitute the roof of the triangle, and the lumbar fascia, internal oblique muscle, and aponeurosis of the transversalis muscle form the floor [3]. See Fig. 1.

TAWH was first described by Doersch and Dozier in 1968 [4] and is usually visualized by CT scans [3]. In a recent metaanalysis CT scans could establish the diagnosis in all but one case (97.7%) [5]. TAWH is a rare injury in emergency rooms, especially in high-velocity road traffic accidents [6]. It is often associated with other injuries, particularly intra-abdominal injuries [7].

The TAWH is often overlooked clinically, together with other abdominal wall injuries such as rectus sheath haematomas and Morel-Lavallée lesions [8]. They are uncommon in a clinical setting, accounting for only 0.17–1.5% of all blunt trauma admissions [9–11]. The most frequent mechanism of injury is a motor vehicle collision [10, 12] and was associated with a deceleration injury from a seat belt [10]. The most common localization is the inferior lumbar triangle (81%) [10] but a TAWH can occur in other locations of the abdominal wall as well [13].

A recent systematic review suggests that early recognition is key in the clinical diagnosis of TAWH, even when it is not the primary life-threatening injury [14].

### Aim of the study

1. To determine the overall incidence of TAWH in a large postmortem collective of trauma cases.
2. To evaluate the specificity of motor vehicle occupant TAWH in accidents by comparing its occurrence across different accident mechanisms.
3. To identify and quantify correlations between the presence of TAWH and other characteristic injury patterns found in motor vehicle occupants.
4. To investigate the specific association between TAWH and seatbelt use to assess its potential as a seatbelt-related injury.

## Methods

A database search was performed in the Institute of Legal Medicine from 1 January 2011 to 31 July 2023 using World Health Organization codes from the Tenth Revision of the International Statistical Classification of Diseases and Related Health Problems (ICD-10) for traffic accidents (codes V01-V99). Patients identified this way were searched for the presence of a postmortem CT in the radiological picture archiving and communication system (PACS). Archiving in the PACS is anonymous and identified only by the autopsy registration number. The patients were reevaluated for the presence of a TAWH in the non-enhanced postmortem CT scan defined as the disruption of muscle and/or fascial layers. The images were also screened for the protrusion of intra-abdominal content without skin breakage. The majority of CT scanning from 2011 to 2019 was done on a Aquillion 64 CT-scanner (Toshiba, Japan) in 2020 on a Somatom Go.All CT-scanner (Siemens, Germany) and from 2021 to 2023 on a Somatom Force CT-scanner (Siemens, Germany). Due to the long time period and the variety of scanner types, various protocols based on the DGRM guidelines [15] were used. The most recent protocol for the Somatom Force CT scanner (Siemens, Germany) had the following parameters: tube current of 250 mAs, tube voltage of 140 kV, and a pitch factor of 0.6. Reconstruction was performed using hard (Br59) and soft (Bf40) kernels with the following parameters: slice thickness of 0.6 mm and an increment of 0.4 mm in a small field of view of 240 mm for the head and neck, and a large field of view of 500 mm for the whole body spiral.

Exclusion criteria was severe thermic abdominal wall damage. The single reader (a radiologist with 20 years’ experience of postmortem CT scans) was blinded to the accident mechanism. Additional parameters were either retrieved from the database of the forensic department (age, sex, height, weight, position in the vehicle, and seatbelt usage) or evaluated in the CT scans. The presence of fractures of the spine, pelvis, ribs or sternum was recorded as well as sacroiliac joint dislocation, hematoma in the subcutaneous fatty tissue, presence of gas or blood in the abdomen. In addition, the implementation of resuscitation measures (defined as chest compressions) was documented if there were indications of this in the case records.

We analysed the accident procedure of all patients with TAWH in a subgroup of motor vehicle occupants (MVOs). Speed, road and collision type were documented.

Statistical analysis was done using SPSS 28.0 (IBM, USA) using Chi-square- and Mann-Whitney U test.

We acknowledge the use of Google’s AI tools (Gemini pro 2.5) for assistance with language editing and manuscript formatting and DEEPL for the translation of several German terms.

## Results

### Study population

**Fig. 2:**
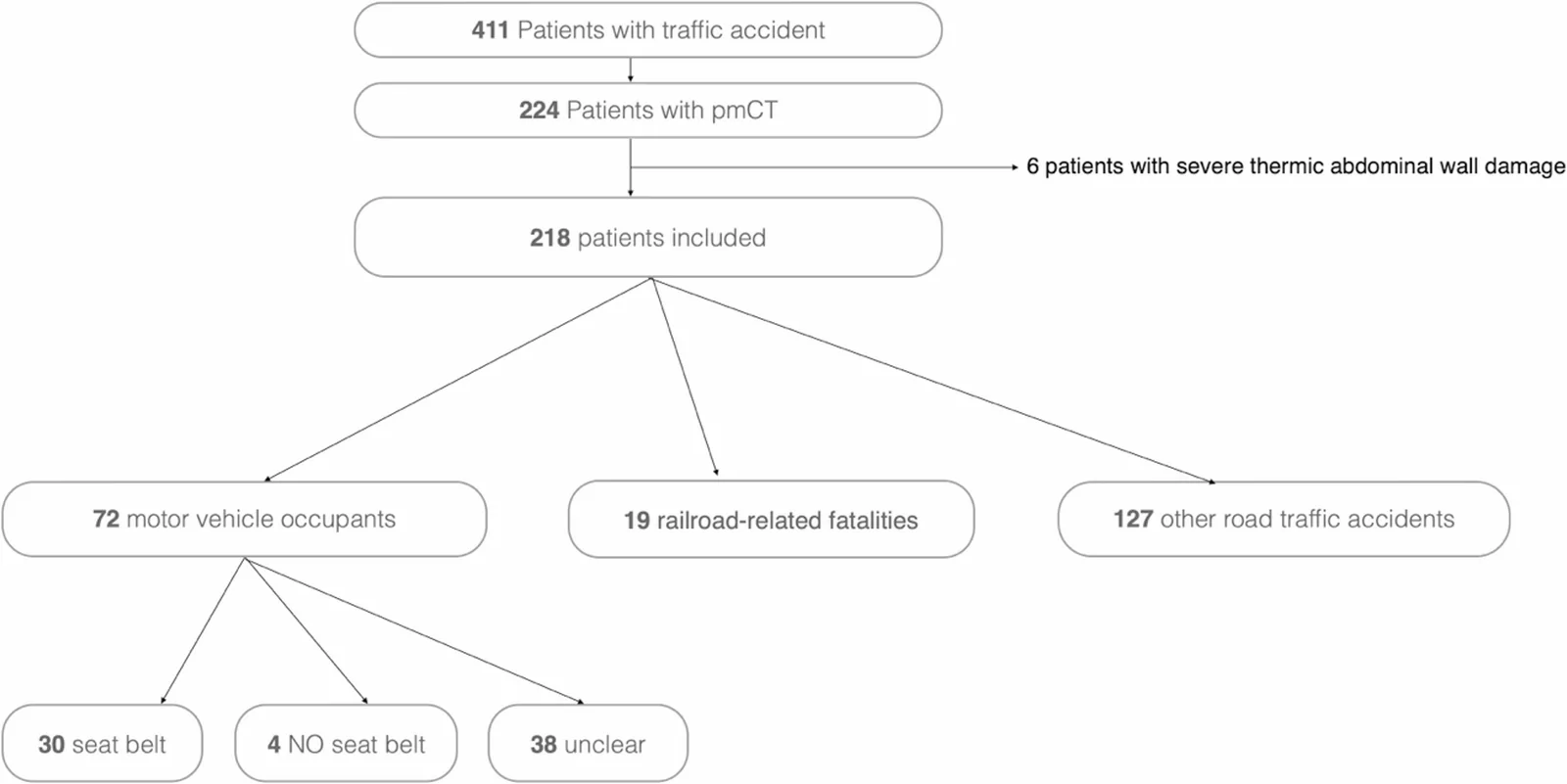
Flowchart of the study population. 6 patients were excluded because of severe thermic destruction of the abdominal wall. The group “other road traffic accidents” included 56 pedestrians, 37 cyclists, 28 motorcyclists and 6 patients with occupying other vehicles like horse-drawn carriages

Of the total number of 411 cases with fatal traffic accidents 224 patients received a postmortem CT-scan, for excluded patients and subgroups see Fig. 2.

### Characterization of study population

The complete study population (218 patients with diagnostic non-enhanced CT scans) consisted of 71 (32.6%) female and 147 (67.4%) male patients. Male patients had an average Body Mass Index (BMI) of 28.4 +- 6.0 kg/m^2^ (*n* = 138), missing data was due to amputation of the lower extremities, dismemberment or decapitation or scale defects. The average age was 50.9 +- 20.2 years (range 2 to 90 years). The female patients had an average BMI of 28.6 +- 7.9 kg/m^2^ (*n* = 69, range 15.5 to 48.8 kg/m^2^) and an average age of 53.8 +-24.9 years (3 to 88 years). BMI and age showed no significant difference between the two sexes. Chest compressions in the course of resuscitation measures were documented in 63 cases (28.9%).

### Incidence

In the total cohort of 218 traffic accident victims, a TAWH was identified in 45 cases (20.6%). The incidence of TAWH did not differ significantly between female patients (19 of 71; 26.7%) and male patients (26 of 147; 17.7%) (*p* = 0.121). Similarly, no significant association was found between TAWH and age group; the incidence was 13.9% (5 of 36) in younger adult patients aged 18–35, compared to 24.0% (17 of 70) in the elderly over 65 (*p* = 0.211). Of the patients with TAWH, only two were under the age of 21 (one aged 17 and one aged 20).

When analyzed by accident mechanism, the incidence was highest among pedestrians in railway collisions, where a TAWH was present in 8 of 19 cases (42.1%). Among these eight patients, the hernia was located on the right side in five cases (26.3%), the left side in one case (5.3%), and was bilateral in two cases (10.5%).

**Fig. 3:**
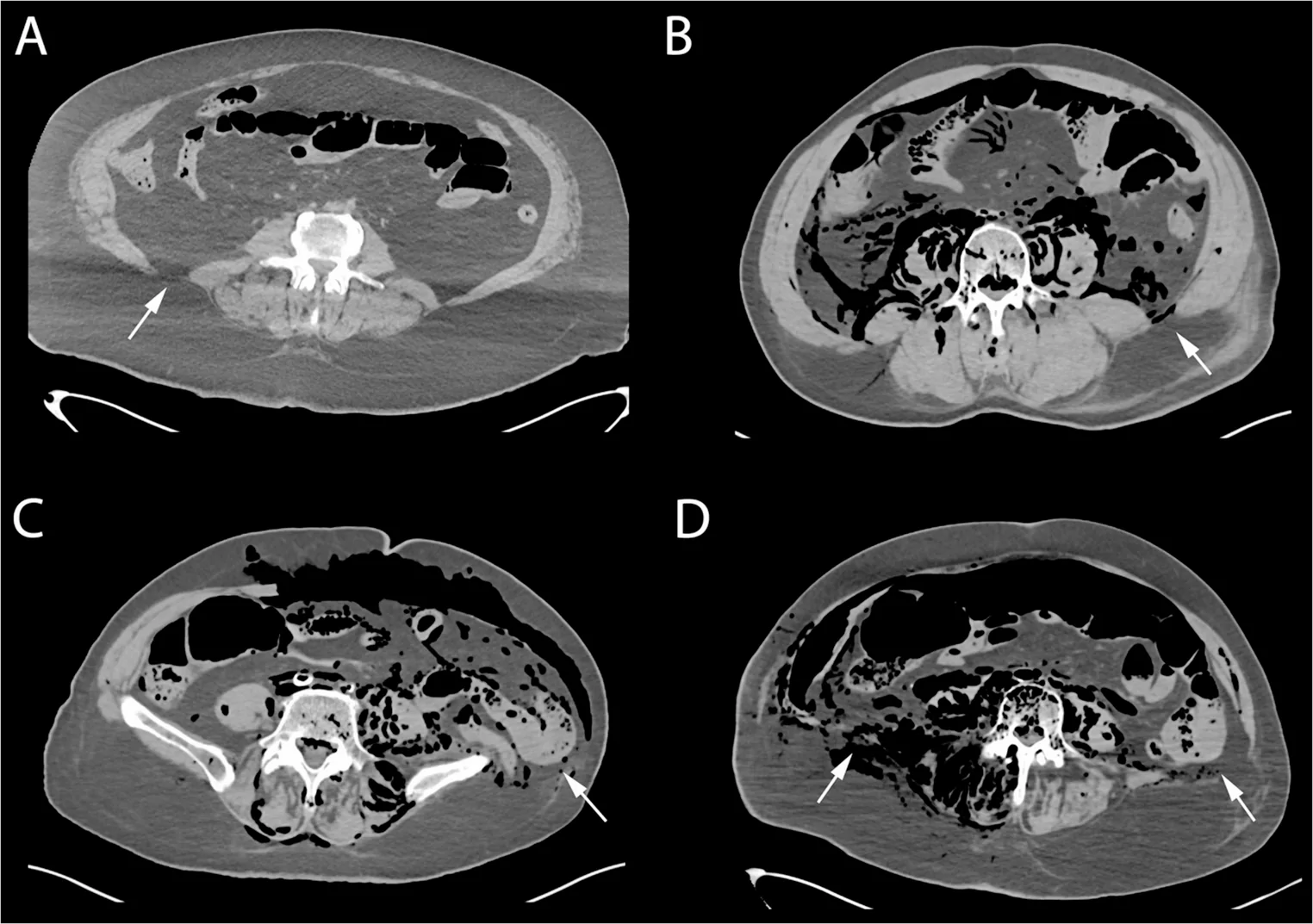
Four axial images of different motor vehicle occupants in a soft tissue window. The pathology is indicated with white arrows. (**A**) pmCT of a right sided TAWH in a 73-year old female motor vehicle occupant seated in the front passenger seat with a BMI 38.5 kg/m². (**B**) pmCT of a left sided TAWH in a 46-year old male motor vehicle occupant seated in the front passenger seat with a BMI 38.5 kg/m² with hematoma in the left flank region. (**C**) pmCT of a left sided TAWH in a 80-year old female motor vehicle occupant seated in the driver’s seat with a BMI 26.5 kg/m² with small bowel loops as content of the hernia. (**D**) pmCT of bilateral TAWH in a 81-year old female motor vehicle occupant seated in the front passenger seat with a BMI of 29.4 kg/m²

Within the MVO subgroup (*n* = 72), the incidence was 19.4% (*n* = 14). Of these, eight were located on the left side (11.1% of all MVOs), four on the right side (5.6%), and two were bilateral (2.8%). In the remaining 127 victims of other traffic accidents, the TAWH incidence was 18.1% (*n* = 23) see Fig. 3.

A weak but statistically significant correlation was observed between the presence of a TAWH and a higher Body Mass Index (BMI) across all accident types (Mann-Whitney U, *p* = 0.049). Patients with a TAWH had a mean BMI of 29.98 kg/m^2^ (SD 6.35, *n* = 42), compared to 28.13 kg/m^2^ (SD 6.72, *n* = 163) for those without.

### Subgroup motor vehicle occupants

From the total cohort of 218 patients, a subgroup of 72 patients was identified as MVO. Within this subgroup, 14 individuals (19.4%) were diagnosed with a TAWH on postmortem CT analysis. None of the MVO with TAWH were younger than 21 years old.

There was no statistically significant association between the occurrence of a TAWH and patient age (Mann-Whitney U test, *p* = 0.071) or sex (Pearson’s χ² test, *p* = 0.123). The mean age for patients with a TAWH was 34.4 years (SD 7.2), compared to 29.8 years (SD 7.0) for those without a TAWH. Chest compressions were documented in 17 patients (23.6%).

Precise information about seatbelt usage could be acquired with certainty in only 34 of the 72 MVOs (47.2%). Of these, 30 patients were wearing a seatbelt and 4 were not. A TAWH was identified in 7 of the 30 seatbelted patients (23.3%), whereas no TAWH was found in the 4 non-seatbelted patients.

Among the 14 patients with a TAWH, seven were drivers and seven were passengers.

- In the driver group (*n* = 7), the TAWH was located on the right side in five cases, the left side in one case, and was bilateral in one case.
- Among the passengers with a TAWH (*n* = 7), the hernia was on the right side in three cases, the left side in three cases, and was bilateral in one case. The seating positions of these passengers were: right front seat (*n* = 4), right rear seat (*n* = 2), and rear middle seat (*n* = 1).

The presence of a TAWH was found to be significantly and positively correlated with several other traumatic injuries, particularly those affecting the pelvic girdle and thoracic cage. The strongest correlation was observed with injuries to the right sacroiliac joint. All statistically significant correlations are detailed in Table 1. Chest compression during resuscitation was considered a confounding factor for rib and sternal fractures. In these cases, the analysis was therefore performed separately on MOV patients without documented chest compressions. (Sternal fracture *N* = 54, *n* = 15, *p* = 0.005; serial rib fractures *N* = 45 *n* = 35 *p* = 0.016)

**Table 1:** Statistically significant correlations between traumatic injuries and the presence of TAWH in Motor Vehicle Occupants, n=Total number of injuries observed, N = Number of cases analyzed, ϕ = phi correlation coefficient. A p-value of less than 0.05 was considered statistically significant and a p-value of less than 0.001 was considered highly significant and shown in bold

Associated injury	*n*	*N*	ϕ	*p*-value
Pelvic injuries				
Right sacroiliac (SI) joint injury	7	72	0.549	**< 0.001**
Left sacroiliac (SI) joint injury	9	72	0.345	0.003
Spinal injuries				
Lumbar transverse process fracture (right)	26	72	0.361	0.002
Lumbar transverse process fracture (left)	36	72	0.351	0.003
Thoracic vertebral body fracture (T5-T8)	14	71	0.379	0.001
Thoracic injuries				
Sternal fracture	20	71	0.419	**< 0.001**
Serial rib fracture	47	71	0.312	0.008

Injuries without a correlation with the occurrence of TAWH are shown in Table 2.

**Table 2:** Correlation between TAWH in motor vehicle occupants and other injuries with no significant correlation. n = number of patients with injuries; N = Number of cases analyzed, ϕ = phi correlation coefficient. For incidences ≤ 5 no statistical correlation was calculated

Associated injury / finding	*n*	*N*	ϕ	*p*-value
Intraperitoneal blood	29	68	0.149	0.224
Clavicular fracture (either side/bilateral)	18	70	0.114	0.346
Sacrum fracture (right)	14	72	0.202	0.089
Sacrum fracture (left)	12	72	-0.031	0.794
Thoracic vertebral body fracture (T1-4)	9	70	0.021	0.861
Thoracic vertebral body fracture (T9-12)	7	72	-0.043	0.721
Ilium fracture (left)	7	72	0.076	0.528
Ilium fracture (right)	5	72	-	-
Lumbar vertebral body fracture (L1-3)	2	72	-	-
Chance fracture	1	72	-	-
Lumbar vertebral body fracture (L4-5)	0	72	-	-

In the MVO with TAWH group’s analysis of the accident procedure, 8 of the 14 accidents were head-on collisions, 3 were side impacts and the remaining 3 had an intermediate angle between head-on collisions and side impacts. Reliable speed documentation was unavailable in 13 out of 14 cases but all accidents occurred on streets outside urban areas suggesting high velocity accidents. All MVO in this subgroup were travelling in a passenger car. Most accidents occurred when two passenger cars collided (9/14); three collisions were with a truck, one with a delivery van, and one with a tree.

## Discussion

This study investigated the incidence and associated injury patterns of TAWH in a postmortem cohort, with a specific focus on MVOs. MVOs happen more frequently than train accidents and are more likely to be survived. Therefore, this patient group seems to be of the greatest interest. Studying this group in further detail and gaining an understanding of the associated injuries and injury patterns could help save lives. The primary finding is that the overall incidence of TAWH in fatal traffic accidents (20.6%) is substantially higher than the 0.17% to 1.5% reported in clinical series of surviving blunt trauma patients [9–11]. This suggests that TAWH may be an under-recognized marker of severe, often lethal, traumatic forces. The incidence was highest among pedestrians struck by trains (42.1%), further underscoring the association of TAWH with extreme high-energy impact mechanisms.

A key objective of this study was to investigate the relationship between TAWH and seatbelt use. While our results showed that all TAWHs with confirmed seatbelt status occurred in belted occupants (7 of 30), a definitive conclusion is precluded by significant data limitations. Seatbelt usage could only be confirmed in 47.2% of cases, and the non-seatbelted group was too small (*n* = 4) for meaningful statistical comparison. However, considering the high rates of seatbelt compliance in Germany—reported at 98.6% for drivers in 2023 [16]—it is highly probable that the majority of patients with undocumented status were, in fact, wearing seatbelts. This would imply that the actual incidence of TAWH in seatbelted occupants is likely higher than what our limited confirmed data shows. This finding aligns with the concept of the “seatbelt sign” and supports the theory that TAWH can result from a deceleration injury where the lap belt acts as a fulcrum, causing a sudden, massive increase in intra-abdominal pressure that leads to fascial and muscular rupture [10]. While TAWH has been reported in unrestrained occupants [3], our data suggests it may be a more characteristic injury in the restrained population.

The strong and statistically significant correlations between TAWH and other specific injuries provide compelling evidence for this proposed mechanism. The association with sternal and serial rib fractures (**ϕ** = 0.419, *p* < 0.001 and **ϕ** = 0.312, *p* = 0.008, respectively) points to significant force application across the chest by the shoulder portion of the seatbelt or the steering wheel. Chest compression in resuscitation was not a confounding factor. Concurrently, the correlation with lumbar transverse process fractures (right: *p* = 0.002, left: *p* = 0.003) and sacroiliac joint injuries (right: *p* < 0.001, left: *p* = 0.003) indicates massive force transmission through the lumbopelvic region, consistent with lap belt loading. Fractures of the transverse processes, in particular, can be caused by avulsion from forceful contraction of the quadratus lumborum muscle during violent hyperflexion over the lap belt. These injury patterns are characteristic of MVO trauma, as described by [6, 17], and their strong correlation with TAWH reinforces the mechanical link between the safety restraint, the resulting force vectors, and the observed abdominal wall failure.

The subgroup of MVO with TAWH was small, and reliable speed documentation was scarce. Therefore, no statistical analysis was performed, although the data suggests the potential for a meaningful analysis of a larger cohort, given that there were no rear-end collisions. In a larger cohort with well-documented speed and accident mechanisms, a correlation between TAWH and accident procedure seems possible. Another possible explanation for the lack of rear-end collisions is that this type of collision is less likely to be fatal than other types of collision [18].

It can be assumed with a high degree of probability that TAWH only occurred at high speeds within this small collective. However, since all of these accidents were fatal, a strong selection bias must be present within the overall collective.

In contrast to these strong mechanical correlations, demographic factors such as age and sex were not significantly associated with TAWH occurrence. A weak but statistically significant correlation with a higher BMI was noted in the overall traffic accident population (*p* = 0.049), but this was not a primary finding within the MVO subgroup analysis. This suggests that while pre-existing factors like a higher BMI might slightly increase susceptibility, the overwhelming determinant for TAWH is the nature and magnitude of the applied traumatic forces. Furthermore, our data showed no clear pattern linking the side of the hernia to the occupant’s seating position; for example, five of seven drivers presented with a right-sided hernia, which is counterintuitive if a simple interaction with the steering column were the cause. This complexity suggests that the injury mechanism is multifactorial, involving a combination of direct impact, seatbelt loading, and inertial forces.

## Limitations

This study has several limitations. First, as a retrospective analysis from a single forensic institute, it is subject to selection and information bias, as not all fatalities from motor vehicle accidents undergo forensic autopsy at our institution. The single center study design also limits the generalizability of the findings. Second, the reliance on police and medical reports for crucial data like seatbelt usage resulted in a substantial amount of missing information, limiting the statistical power of our seatbelt analysis. Third, the diagnosis on non-enhanced postmortem CT scans did not allow for the detailed grading of hernias according to classifications like [11], as Grade III injuries involving muscular disruption without herniation of content can be difficult to distinguish from hematomas. Fourth, the study design only used a single reader for the positive diagnosis of lesions, thus preventing the calculation of inter-observer variability. Finally, the relatively small number of TAWH cases (*n* = 14) within the MVO subgroup restricts the generalizability of the findings regarding seating position and specific injury patterns.

## Conclusion

In this postmortem cohort, TAWH was found at a much higher incidence (20.6%) than is reported in clinical literature, identifying it as a significant marker of fatal, high-energy trauma. TAWH is not specific to motor vehicle occupants but is strongly associated with injury patterns characteristic of seatbelt loading, including thoracic cage, lumbar spine, and pelvic injuries. While our data strongly indicates a correlation between seatbelt use and TAWH, the small number of confirmed non-wearers prevents a definitive conclusion. These findings underscore the need for a high index of suspicion for TAWH in severely injured trauma patients, particularly those with a “seatbelt sign” or associated fractures. Further biomechanical research is warranted to precisely define the injury thresholds and mechanisms leading to traumatic abdominal wall failure.

## Data Availability

The datasets generated during the current study are available from the corresponding author on reasonable request.
